# Modelling performance by continents in swimming

**DOI:** 10.3389/fphys.2023.1075167

**Published:** 2023-05-23

**Authors:** I. Yustres Amores, J. Santos del Cerro, F. González-Mohíno, F. Hermosilla, J. M. González-Ravé

**Affiliations:** ^1^ Facultad de Ciencias de la Salud, Universidad Francisco de Vitoria, Madrid, Spain; ^2^ Department of Applied Economics I, University of Castilla-La Mancha, Toledo, Spain; ^3^ Sport Training Laboratory, University of Castilla-La Mancha, Toledo, Spain; ^4^ Facultad de Ciencias de la Vida y de la Naturaleza, Universidad Nebrija, Madrid, Spain; ^5^ Department of Physical Activity and Sports Science, Alfonso X El Sabio University, Madrid, Spain

**Keywords:** modelling, performance, continent, swimming, analysis

## Abstract

**Introduction:** There is a growing interest in the scientific community about the progression and congruity in the performance of talented participants who complete representing different nations in the most important international events. The prediction of incoming performances is nowadays in demand with the objective of returning in talent investment. Talent identification programs have tried to select and develop sports talent over years. However, to our knowledge, there is a lack of research about success in swimming World Championships (WCs) performance considering continents-country and how successful outcomes are influenced by these variables. Therefore, the primary goal is to analyze the effect of early specialization comparing the performance progression model of the countries gathered by continents.

**Methods:** Participant’s data from all Junior and Senior WCs between 2006 and 2017 from International Swimming Federation (FINA). One-way ANOVA, ANCOVA and regression model were used to explain whether the variable category, age, best z-score, experience, and continent influences the performance obtained in Absolute WC.

**Results:** Significant differences (*p* < 0.01) were found between the average performance obtained by the two different categories (junior: swimmers participating in junior WCs before senior WCs; senior: swimmers participating in senior WCs without previous participation in junior WCs), where swimmers from category junior showed significant better performance’s times than seniors, except in America. ANCOVA results showed that generally, the greatest differences where in the earliest ages, with best performance registered in category junior in all the continents. Also, the experience was a significant variable in the general model.

**Conclusion:** Swimmers who had participated in junior category prior absolute obtained better performance’s times than those swimmers who participated directly in absolute, in the first participation in senior WC. Thus, early specialization is a key factor to obtain better results in senior WCs for all the continents, except in America.

## 1 Introduction

There is a growing interest in the scientific community about the progression and congruity in the performance of talented participants who compete representing different nations in the most important international events (Olympic Games [OG] and WCs) (Yustres et al., 2017; Yustres et al., 2019a; Yustres et al., 2019b).

Specifically, in swimming, several investigations measured the variation in performance and characterization of elite swimmers from different countries amongst the best international competitions over the years due to be considerate an important factor for tracking the performance progression (Yustres et al., 2017; Yustres et al., 2019a; Yustres et al., 2019b). An important predictor of the successful in senior category were the fast times of swimmers at the junior WCs (Yustres et al., 2019b), but not the finalist positions (Yustres et al., 2017). However, these authors found a strong association (*p* < 0.001) between the final position in WCs and maintenance years, showing that each subsequent WCs that swimmers compete in, they get 3.02 higher ranking positions (Yustres et al., 2017; Yustres et al., 2019b). These results indicate that the experience in major championships has an impact on a better finishing position, suggesting starting the competition at higher levels at an early age. In this sense, it has been also found that swimmers performing in junior WCs had more chances to achieve posteriori success in senior WCs (Yustres et al., 2019b). In addition, those swimmers with more years competing at WCs had a positive impact to achieve better positions. The progression between and within competitions has been shown as a key variable to success in major championships in elite swimmers (Pyne et al., 2004). In this sense, most nations scrutinize medal performances at different status because they have invested a lot of money in developing the best sporting systems and infrastructures to recognize and develop exceptionally talented athletes for obtaining medals (Seiler, 2013).

The measurement of competitive performance variables, such as time, distance, scores, is affected by different variables as physiological and skill demands of the sport, dynamics of competition, environmental and other external factors affecting performance, and the measure of the competitive performance itself (Seiler, 2013; Malcata and Hopkins, 2014). The performance analysis of these athletes will allow analyzing the relationship between world-ranking countries and how successful outcomes are influenced by these variables (Seiler, 2013; Malcata and Hopkins, 2014).

The longitudinal evaluation of seasons or/and international competitions could therefore be defined by measuring the swimmers’ results for a specific period, analyzing their progression in performance, and helping coaches to develop realistic goals and to select appropriate training methods (Pyne et al., 2004; Costa et al., 2010). Therefore, these are essential items to consider when evaluating the performance of a nation (Seiler, 2013). As a complement to the study of differentiated performance by countries, recent studies are also conducting research on the differences between continents to find out if there is any differentiated pattern between them.

The FINA WC is the competition in which each continent is represented by the best swimmers who participate as representatives of the different countries that comprise it.

There is a current interest in analyzing the effect of the variable’s country and continent in different aspects and sports. An important investigation analysed performance and participation trends in “Ironman Hawaii” examining the country and continent of the participants that crossed the final line, concluding that was dominated by United States of America in both genders, both in participation and performance (Cejka et al., 2013). A similar study analysed the participation and performances of 21,399 athletes at the ‘Ironman Switzerland’ regarding the country. Concluding that both competition and performance were dominated by central European triathletes (Jürgens et al., 2012). Also, performance and participation trends in duathletes in the ‘Powerman Duathlon World Championship’ regarding the country of the finishers, was examined. Results showed that European athletes dominated participation in the ‘Powerman World Championship’ (Rüst et al., 2013).

However, to our knowledge, there is a lack of research about success in swimming WC performance considering continents-country and how successful outcomes are influenced by these variables. That’s the reason why the aim of the study is to examine the relationship between world-ranking countries categorized by continents and WC performance. Secondary goals included analyzing the effect of early specialization comparing the performance progression model of the countries gathered by continents.

## 2 Materials and methods

### 2.1 Subjects

Data (29,031 entries) were obtained from FINA (http://www.fina.org/).

All historical data was retrieved from the results of the official website for the 2006, 2008, 2011, 2013, 2015, and 2017 junior WCs and 2007, 2009, 2011, 2013, 2015 and 2016 senior WCs. Final database contained 29,031 entries. After removing duplications, database was formed by records from 5,878 swimmers.

The final filtered database included only swimmers whose age in the competition is equal to or greater than 16 years and data were recorded as mean (±standard deviation, SD) by category, distances, gender, country, continent, year of competition and swim strokes for a more appropriate standardization of the times.

Then, the final database was divided into two categories: swimmers who have at least one participation in junior competition and then have competed in absolute category (junior) and those who have only participated in absolute category (absolute).

The variables showed for each entry are the following: country, status [highest finishing position: final (3), semifinal (2), heats (1)], race time, age, distance, gender, swim stroke, maintenance years (number of participations in WCs), continent and the year of competition. 200 and 400 m individual medleys: 50, 100, and 200 m backstroke/breaststroke/butterfly and 50, 100, 200, 400, 800, and 1500 m freestyle are the distances analysed.

This retrospective study was conducted in accordance with the declaration of Helsinki. Since the data are based on publicly available resources, no informed consent was obtained.

### 2.2 Procedure

Times have been standardized by means of Z-time scores to compare swimmers’ times without influencing distance, swim stroke, category, and gender. To include the annual best result of each swimmer, Z-time was adjusted categorized by distances, swim stroke, and gender. Besides, to make the comparisons as homogeneous as possible, age has been referenced in each of the variables constructed.

$$Z_{ij}=\frac{X_{ij}-{\bar{X}}_{i}}{\sigma_{i}}$$

Where *i* = group, *j* = individual by distance, swim stroke and gender.

The following variables have been defined: best z-score in the first absolute competition; annual average progress of the second, third, fourth and fifth participation as absolute with respect to the first participation as absolute. The criteria followed to choose the countries that are included in the final database was to develop an overall ranking with the whole number of nations participating in each one of the WCs from 2006 to 2017. In this sense, information about the ranking points for each WCs was searched on internet, and an overall ranking was created combining all this information and removing those countries that did not compete in all the WCs analysed. A total of 124 countries divided between the 5 continents had participated in all the WCs analysed in this investigation.

### 2.3 Statistical analysis

One-way ANOVA was used to assess whether the Category (junior and absolute) influences the performance obtained in the first participation in Absolute World Championships. The age was restricted between 17 and 21 years, both inclusive, since for the junior Category the sample size decreases significantly from the age of 22 and there were not enough data to carry out a rigorous analysis.

Besides, ANCOVA was carried out with the variable age added as covariable. The ANCOVA analysis was repeated for each of the continents analysed to assess whether the Category variable and its interaction with the covariable age influence in the performance obtained in the first participation.

Adding, a regression model that explains best_z_per (the best z-score of the year for each swimmer in percentage), regarding their age, category (absolute and junior) as well as their absolute competition experience measured by the number of accumulated participations in that type of competition was carried out. A general or global model was performed, and the same model was replicated for each continent. The variable Category was defined by assigning the value 0 to the absolute category and 1 to junior.

All analyses were performed with the R software. The level of significance was set at *p* ≤ 0.05 was the level of significance.

## 3 Results

Firstly, a general analysis of all swimmers will be shown to know the sample used and its behaviour. Then, a more exhaustive examination of their performance will be carried out by continent. The number of swimmers by age and by category (junior versus absolute) from all the countries analysed in their first participation in Absolute WCs are shown in Table 1, being Europe (226) and America (148) the continents which provided a higher number of participants in junior, meanwhile in absolute changed to Europe and Asia (1,023 and 662 respectively). Besides, the highest participation percentage for both (swimmers who participate directly in absolute WC and those who have previously participated in junior) is established at 19 years (12.3% and 25.5% respectively).

**TABLE 1:** Number of participants in WC (2006–2017).

Continent		Age at first participation in absolute WC												
		16	17	18	19	20	21	22	23	24	25	26		Total
JUNIOR	Africa	0.0%	20.5%	35.9%	12.8%	17.9%	10.3%	2.6%	0.0%	0.0%	0.0%	0.0%	100.0%	39
	America	0.0%	12.2%	25.0%	24.3%	17.6%	8.8%	2.7%	4.7%	1.4%	2.0%	1.4%	100.0%	148
	Asia	0.0%	16.7%	22.2%	25.0%	13.9%	5.6%	11.1%	5.6%	0.0%	0.0%	0.0%	100.0%	36
	Europe	0.0%	11.1%	25.2%	26.5%	16.4%	11.1%	3.1%	3.1%	2.2%	1.3%	0.0%	100.0%	226
	Oceania	0.0%	11.5%	3.8%	42.3%	19.2%	11.5%	7.7%	3.8%	0.0%	0.0%	0.0%	100.0%	26
	Total	0.0%	12.6%	24.6%	25.5%	16.8%	9.9%	3.8%	3.6%	1.5%	1.3%	0.4%	100.0%	475

Continent		Age at first participation in Absolute WC												
		16	17	18	19	20	21	22	23	24	25	26		Total
ABSOLUTE	Africa	13.6%	16.7%	13.2%	14.0%	8.1%	10.1%	7.4%	3.9%	7.4%	4.3%	1.6%	100.0%	258
	America	7.1%	9.1%	8.9%	8.5%	13.6%	10.5%	14.9%	8.9%	7.8%	7.1%	3.6%	100.0%	449
	Asia	11.0%	9.4%	15.6%	15.0%	10.0%	10.0%	8.9%	7.9%	6.3%	3.0%	3.0%	100.0%	662
	Europe	5.6%	9.5%	7.1%	12.4%	12.2%	13.6%	12.5%	10.9%	7.3%	5.2%	3.7%	100.0%	1,023
	Oceania	16.3%	14.1%	8.2%	8.7%	10.3%	13.0%	9.8%	6.5%	5.4%	4.9%	2.7%	100.0%	184
	Total	8.8%	10.4%	10.3%	12.3%	11.3%	11.7%	11.3%	8.7%	7.0%	4.9%	3.2%	100.0%	2,576

**FIGURE 1:**
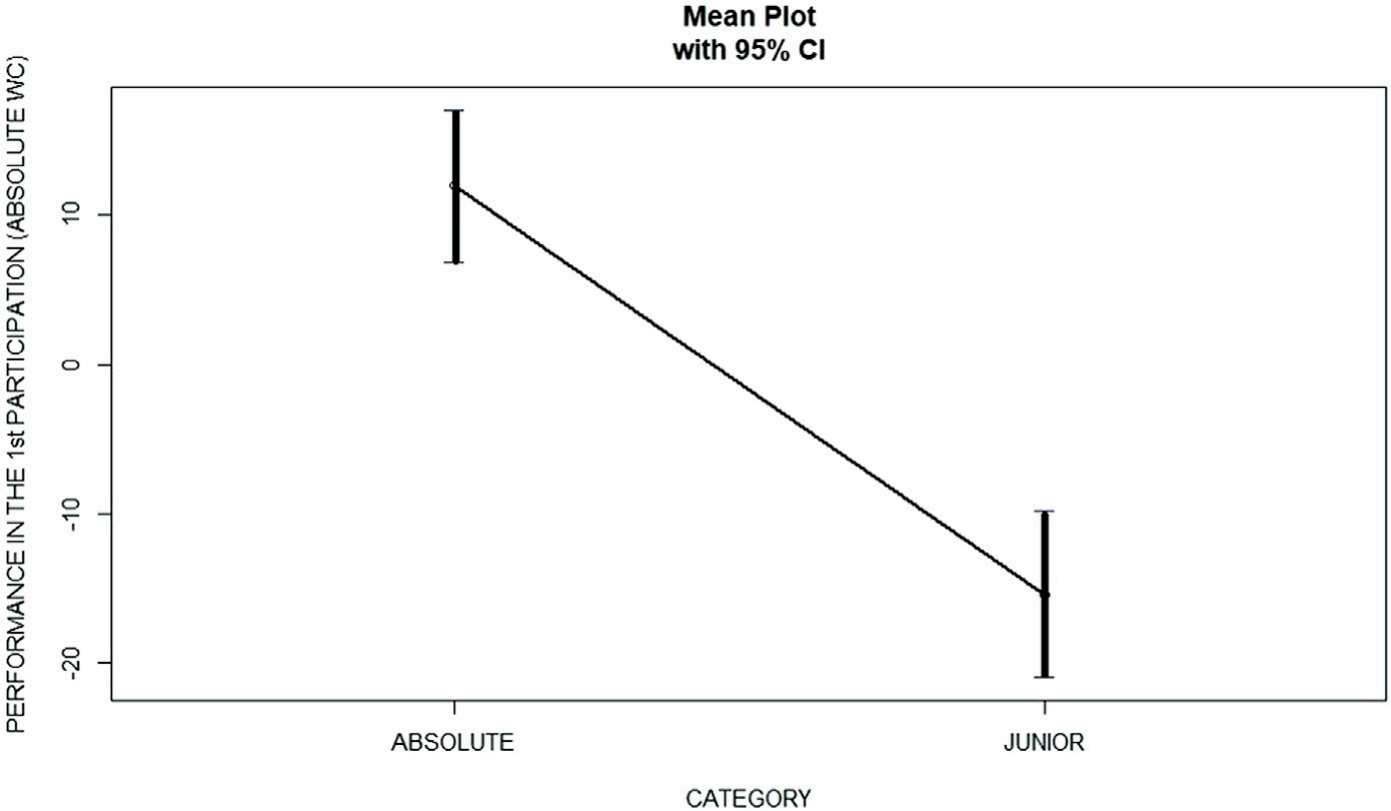
Differences in perfomance (Zscore) in the 1st participation in absolute WC.

One-Way ANOVA focusing on whether the category influences the performance obtained in the first participation in WCs was carried out. Significant differences (*p* < 0.01) were found between the average performance obtained by the two different categories (Table 2) where swimmers from category junior showed significant better performance’s times than absolute swimmers (Figure 1).

**TABLE 2:** Anova one-way.

	Df	Sum sq.	Mean sq.	F Value	Pr (>F)
Category	1	245,930	245,930	29.73	5.64e-08 ***
Residuals	68	15,454,212	8,273		

Signif. codes: 0 ‘***’ 0.001 ‘**’ 0.01 ‘*’ 0.05 ‘.’ 0.1 ‘ ’ 1.

**FIGURE 2:**
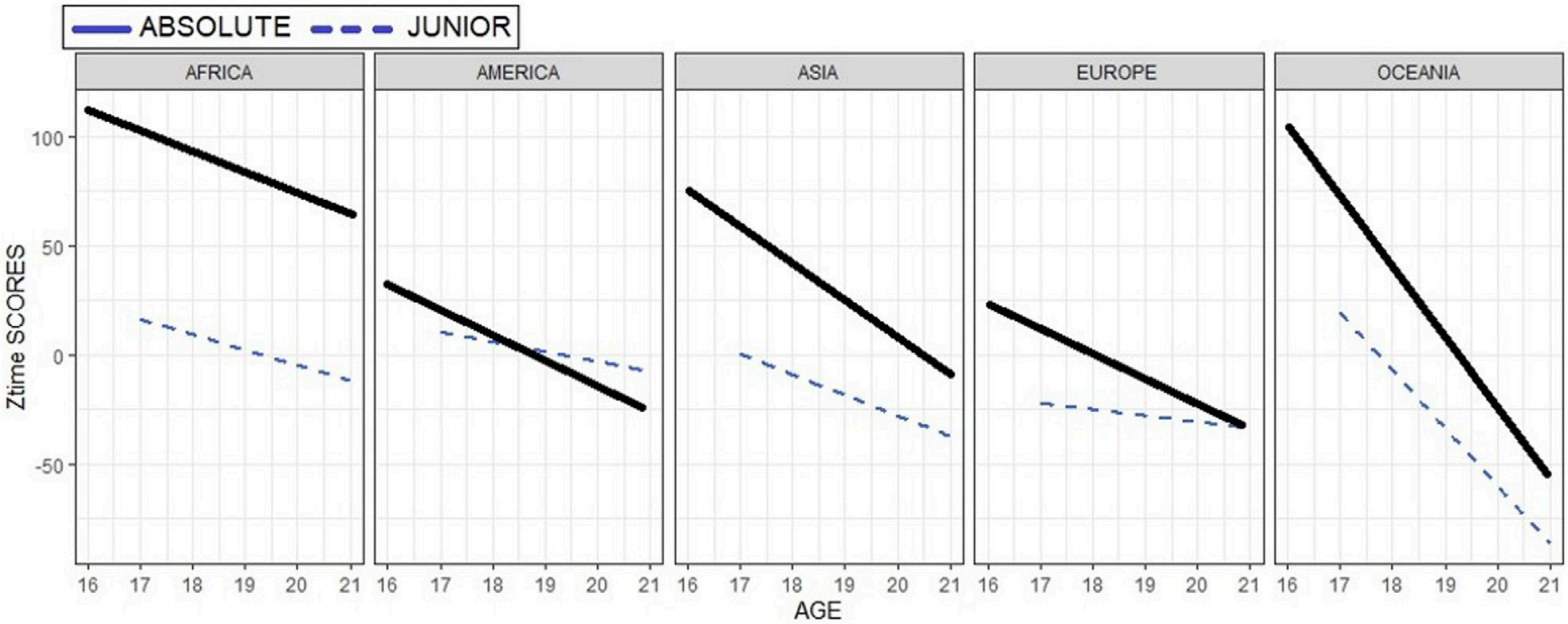
Perfomance (Zscore) according to age by continents and category.

The previous model was extended to an ANCOVA in which the age was added as covariable. The analysis was repeated for each of the continents analysed to assess whether the category variable and its interaction with the covariable age influence in the performance obtained in the first participation. Results showed that both the individual and cross effects were significant. Besides, graphically, it has been shown that the difference in performance between the two categories tends to converge with age as shown in the following figure (Figure 2).

**FIGURE 3:**
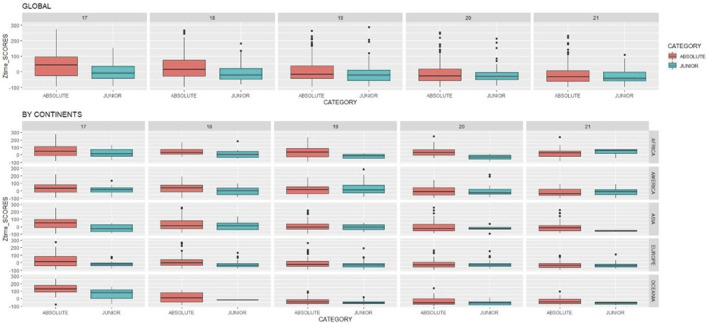
Global and continental differences in perfomance (Zscore) by category and age.

Also, when analyzing the differences in performance obtained at the first participation by category and age, results showed how globally, swimmers who have participated previously in junior category obtained greater performance than those who just participated straight to absolute category. Specially in early ages as it is shown in global results in Figure 3.

The analysis was repeated for each continent. Results again confirmed that generally, the greatest differences in the earliest ages with best performance registered in swimmers who had previously participated in junior category before participating in absolute against those swimmers who directly performed in absolute category in all the continents, as shown in by continents results in Figure 3. Showing that the highest different between the performance obtained by category are shown for Europe and Oceania.

The general model showed how, all the linear regression models were significant measured through the F contrast. Likewise, the category variable was significant with a negative value in all the models except in the one corresponding to the American continent (Table 3). European swimmers showed that their mark (expressed according to the z-score in percentage) was 10% better for junior than for absolute. The age variable, expressed in years, was significant in all models except in Africa due to a more heterogeneous behavior of their swimmers. In general, for each year that swimmers meet, their percentage z-score improves by approximately 5%. Regarding the number of participations in absolute championships for each swimmer, although in the global model it is significant, in the models by continents it is not so in Europe, America and Oceania.

**TABLE 3:** Global and continental lineal model.

Global lineal model			
Coefficients	Estimate	*p*-value of estimate coefficient	Global significance (F-statistic): *p*-value
Intercept	125.39	<2e-16	<2.2e-16
Category	−19.47	3.43e-09	
Age	−5.75	<2e-16	
Participations	−5.34	7.93e-05	
Europe lineal model			
Coefficients	Estimate	*p*-value of estimate coefficient	Global significance (F-statistic): *p*-value
Intercept	68.68	<2e-16	<2.2e-16
Category	−10	0.00279	
Age	−4.27	<2e-16	
Participations	−2.39	0.07693	
America lineal model			
Coefficients	Estimate	*p*-value of estimate coefficient	Global significance (F-statistic): *p*-value
Intercept	103.12	5.18e-14	<2.2e-16
Category	15.38	0.00304	
Age	−5.79	<2e-16	
Participations	0.82	0.73719	
Asia lineal model			
Coefficients	Estimate	*p*-value of estimate coefficient	Global significance (F-statistic): *p*-value
Intercept	137.82	9.13e-16	7.15e-15
Category	−44.02	0.00075	
Age	−5.25	1.84e-08	
Participations	−9.92	0.00690	
Africa lineal model			
Coefficients	Estimate	*p*-value of estimate coefficient	Global significance (F-statistic): *p*-value
Intercept	99.09	0.00269	1.70e-09
Category	−68.1	0.00030	
Age	1.46	0.37399	
Participations	−42.43	5.6e-08	
Oceania lineal model			
Coefficients	Estimate	*p*-value of estimate coefficient	Global significance (F-statistic): *p*-value
Intercept	259.73	<2e-16	<2.2e-16
Category	−46.41	0.00091	
Age	−13.1	<2e-16	
Participations	8.94	0.09759	

## 4 Discussion

The main objective of the study was to analyze the relationship between world-ranking countries categorized by continents and WC performance. Secondary goals included analyzing the effect of early specialization comparing the performance progression model of the countries gathered by continents.

The most important findings were that generally, swimmers from junior obtain better performance’s times than absolute swimmers in the first participation in WCs, except in America. Age is a key factor in America since swimmers are 19 years old. Swimmers improve their personal best more in absolute than juniors. The American governing body is organized through a pyramid system where only is possible to achieve the top (high performance), delivering the best-in-class in world swimming results. Only a few countries (i.e., the United States, China, Great Britain, Germany, etc.) have the challenging combination of large population, diverse sports culture, rich economy, favourable seasonal conditions, national coaching expertise, and well-developed facilities infrastructure to make themselves podium candidates in almost all Summer Olympics sports (swimming included) (Seiler, 2013). These results are in line with the study of Yustres et al. (2019a) were results showed that swimmers achieving an optimal performance at junior WCs from European had a better chance of success at the senior WCs (Yustres et al., 2019a). It has also been found that race performance at junior level is a powerful predictor of successive achievement in senior elite cyclists (Svendsen et al., 2018). Besides, the highest different between the performance obtained by category was found in Europe and Oceania. European swimmers showed that their mark (expressed according to the z-score in percentage) was 10% better for junior than for absolute. This might be justified with an advantage in practice, an already established organization of elite sport associations and existing ‘role models’ for hoping future competitors (Dähler and Rosemann, 2014). The outcomes of a professional sport system are normally measured in terms of performance at the OG for each country, and in a continent, you can find out different country’s success in elite sports, depending on different factors such as genetic predisposition, family support, sport policies or country’s wealth.

Another important finding was that countries with the highest number of swimmer’s participation in absolute WC were Europe and Asia, being Europe and America the continents which provided a higher number of participants in Junior. A possible explanation for the dominance could be the number of qualifying events in each continent (Seiler, 2013; Dähler and Rosemann, 2014). The population of the different countries might also be a reason [5, 20]. In this sense, our results showed how Oceania and Africa are the countries with less swimmer’s representation in both categories. Besides, Asia shows just 36 swimmers participating in junior category. Adding, swimming federations may enhance the performance achievement by targeting resources towards larger groups of swimmers some years out from an international event [20]. Socioeconomic factors and sports systems could be one of the reasons for the limited data that exists. In addition, these results tailored with all-time 10 countries medal table in WC since 1973, first country in the rank is United States (America), second is Australia (Oceania) fourth is China (Asia) and the rest of countries belong to Europe (seven countries).

An important study investigated the origin of swimmers competing in elite open-water competition of all FINA 10 km swimming competition held between 2008 and 2012 (Vogt et al., 2013). Concluding that the country where is held a competition may influence the popularity of this sports, and consequently, the number of participants. Another study analyzed the performance and participation by country at the Ironman Switzerland concluding that 90% of the participants were from European countries (Jürgens et al., 2012). Also showing where the competition take place and therefore small travel distance for local athletes seems to play an important role in the decision for participating at a competition (Jürgens et al., 2012). Besides, it has been concluded that the greater number of European athletes competing in 100-km ultra-marathons was influenced by a mayor popularity of ultra-marathons in Europe in comparison with other continents (Cejka et al., 2013). In this sense and considering our results, it could be interesting analyze the location of the sport event to shed light on such a small athlete’s participation in junior category in Asia as well as in both categories for Oceania and Africa.

On the other hand, our results showed as the variable age was significant in all continents except for Africa. Each year that swimmers meet, their percentage of z-score improves by approximately 5%. Besides, the highest participation percentage for both (swimmers who participate directly in absolute WC and those who have previously participated in junior) was established at 19 years. This is in line with a study where it was found that top swimmers at the age of 17–18 years from America were not ranked in the country’s top-100 at younger ages (Larsen, 2003). This fact could glimpse the different sports policy at an early age in Africa compared to other continents. As it was established in the study of Larsen (2003) the influence of nationality and ethnic background on performance is best known from East-African distance runners (Larsen, 2003). However, in swimming, Africa showed a more heterogeneous behavior of their swimmers with the associated problem this has for their progress and performance.

Besides, regarding the number of participations in absolute championships for each swimmer, the global model showed that as many numbers of participations, the better swimmers’ results obtained. These results are in line with an investigation where the variable position improvement as swimmers attended more WCs (Yustres et al., 2019a). Therefore, an early sports consideration of the most outstanding swimmers to get a greater number of participations could be an interesting strategy for different countries and continents. On the contrary, performance and participation trends in duathletes in the “Powerman Duathlon WC” from 2002 to 2011, showed that athletes from the five countries with the highest participation showed no changes in overall race time (Jürgens et al., 2012). Some limitations need to be acknowledged. Due to the specificity of the statistical analysis, we only compare the age and category (absolute and junior) among the continents, and we do not analyze other variables such as sex, stroke, and distance.

In conclusion, Europe and America are the continents with the highest number of swimmers participating in junior WCs before absolute WC meanwhile in Absolute changed to Europe and Asia. The biggest percentage of participation is established at 19 years in both categories. Swimmers from junior obtain better performance’s times than absolute swimmers in the first participation in World Championships. This ddifferences in performance between the two categories tends to converge with age, being the highest different between the performance obtained by category in Europe and Oceania. European swimmers showed that their performance was 10% better (expressed according to the z-score in percentage) for junior than for absolute. In general, for each year that swimmers meet, their percentage z-score improves by approximately 5%.

## 5 Practical applications

From a practical viewpoint, these conclusions can be used by high performance centers, national or international coaches, clubs, on the results obtained at early ages in the junior category, which may be helping to meet talented swimmers with possible aspirations in the future to follow on that level. Or of already absolute swimmers to know the progress that has been obtained through out of time as a form of motivation to continue that path. Obtaining the key differences for each continent.

Information to help coaches, federations, and high-performance centers to know the risks that early specialization can cause, by dedicating many hours at an early age. It may be useful to know the problems and be able to get the maximum talent out of swimmers from an early age, as well as the benefits that can be achieve by doing several sports at the same time at an early age.

## Data Availability

The datasets presented in this study can be found in online repositories. The names of the repository/repositories and accession number(s) can be found below: https://www.fina.org/swimming/results?year=2022&month=latest&disciplines=SW and omegatiming https://www.omegatiming.com/sports-timing-live-results.

## References

[B1] Allen S. Vandenbogaerde T. Hopkins W. (2014). Career performance trajectories of olympic swimmers: Benchmarks for talent development. Eur. J. Sport Sci. 14, 643–651. 10.1080/17461391.2014.893020 24597644

[B2] Cejka N. Rüst C. A. Lepers R. Onywera V. Rosemann T. Knechtle B. (2013). Participation and performance trends in 100-km ultra-marathons worldwide. J. Sports Sci. 32, 354–366. 10.1080/02640414.2013.825729 24015856

[B3] Costa M. Marinho D. Reis V. Silva A. Marques M. Bragada J. (2010). Tracking the performance of worldranked swimmers. J. Sports Sci. Med. 9, 411–417. –417 PMID: 24149635. 24149635 PMC3761712

[B4] Dähler P. Rosemann T. (2014). Nation related participation and performance trends in 'Ironman Hawaii' from 1985 to 2012. BMC Sports Sci. Med. Rehabil., Med. Rehabilitation 6, 16. 10.1186/2052-1847-6-16 PMC4006525 24735524

[B5] Jürgens D. Knechtle B. Rüst C. A. Knechtle P. Rosemann T. Lepers R. (2012). An analysis of participation and performance by nationality at ‘Ironman Switzerland’ from 1995 to 2011. J. Sci. Cycl. 1 (2), 10–20.

[B6] Larsen H. B. (2003). Kenyan dominance in distance running. Comp. Biochem. Physiol. 136, 161–170. 10.1016/s1095-6433(03)00227-7 14527638

[B7] Malcata R. Hopkins W. (2014). Variability of competitive performance of elite athletes: A systematic review. Sports Med. 44, 1763–1774. 10.1007/s40279-014-0239-x 25108349

[B8] Pyne D. Trewin C. Hopkins W. (2004). Progression and variability of competitive performance of Olympic swimmers. J. Sports Sci. 22 (7), 613–620. 10.1080/02640410310001655822 15370491

[B9] Rüst C. Knechtle B. Knechtle P. Lepers R. (2013). The aspect of nationality in participation and performance at the ‘powerman Duathlon world championship’ – the ‘powerman zofingen’ from 2002 to 2011. J. Sci. Cycl. 2 (1), 33–39.

[B10] Seiler S. (2013). Evaluating the (your country here) olympic medal count. Int. J. Sports Physiology Perform. 8, 203–210. 10.1123/ijspp.8.2.203 23428493

[B11] Sokolovas G. Vilas-Boas J. P. Alves F. Marques A. (2006). Analysis of USA swimming’s all-time top 100 times. Biomechanics and Medicine in Swimming X. Rev. Port. Cien. Desp. 11, 315–317.

[B12] Svendsen I. Tønnesen E. Tjelta L. ØrnTraining S. (2018). Training, performance, and physiological predictors of a successful elite senior career in junior competitive road cyclists. Int. J. Sports Physiology Perform. 13, 1287–1292. 10.1123/ijspp.2017-0824 29745739

[B13] Vogt P. Rüst C. Rosemann T. Lepers R. Knechtle B. (2013). Analysis of 10 km swimming performance of elite male and female open-water swimmers. SpringerPlus 2, 603. 10.1186/2193-1801-2-603 24324922 PMC3853191

[B14] Yustres I. Martín R. Fernández L. González-Ravé J. M. (2017). Swimming championship finalist positions on success in international swimming competitions. PLoS One 12 (11), e0187462. 10.1371/journal.pone.0187462 29108018 PMC5673220

[B15] Yustres I. Santos Del Cerro J. González-Mohíno F. Peyrebrune M. González-Ravé J. M. (2019a). Comparing the pathway to success in European countries competing in the swimming world championships. Front. Psychol. 10, 1437. 10.3389/fpsyg.2019.01437 31297075 PMC6607921

[B16] Yustres I. Santos del Cerro J. Martın R. Gonzalez-Mohıno F. Logan O. González-Ravé J. M. (2019b). Influence of early specialization in worldranked swimmers and general patterns to success. PLoS ONE 14 (6), e0218601. 10.1371/journal.pone.0218601 31220159 PMC6586317

